# Supplementary material for: NopC/T/L Signal Crosstalk Gene GmPHT1-4
Source: Int J Mol Sci. 2023 Nov 20;24(22):16521. doi: 10.3390/ijms242216521 (PMC10671193; doi:10.3390/ijms242216521)
Supplement: Supplementary file 1 [file ijms-24-16521-s001.zip › ijms-2674064-supplementary.pdf]

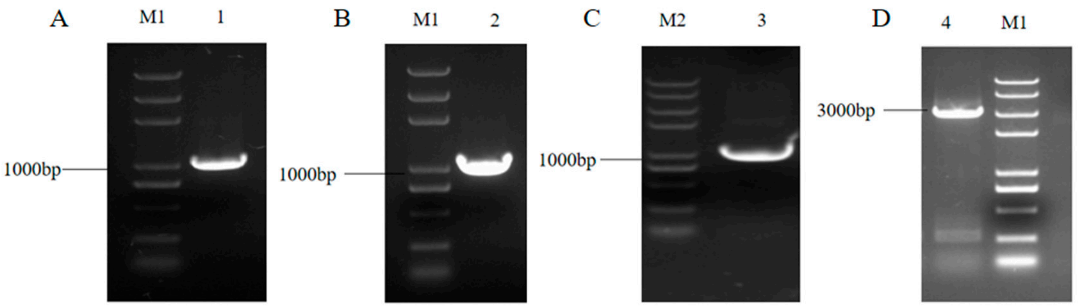

Figure S1. *NopT* upstream and downstream fragments，*Cm* fragments and *NopT-Cm* fragments were amplified. A: upstream fragment of NopT (1020 bp); B: NopT downstream fragment (1000 bp); C: Cm fragment (1010 bp); D: Large fragment of NopT-Cm (3030 bp); M1: Trans 2K Plus DNA marker; M2: Trans 2K PlusII DNA marker.

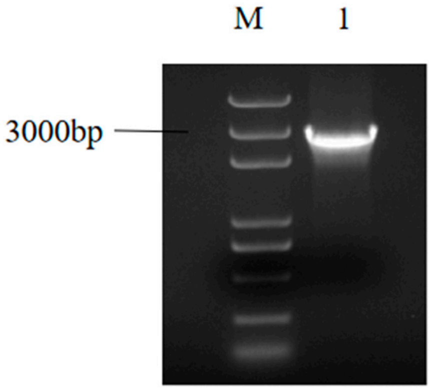

Figure S2. Bacterial fluid PCR for *NopT-Cm*-pJQ200SK. M: Trans 2K Plus DNA marker; 1: Insert a large fragment of *NopT-Cm* of pJQ200SK (3030 bp).

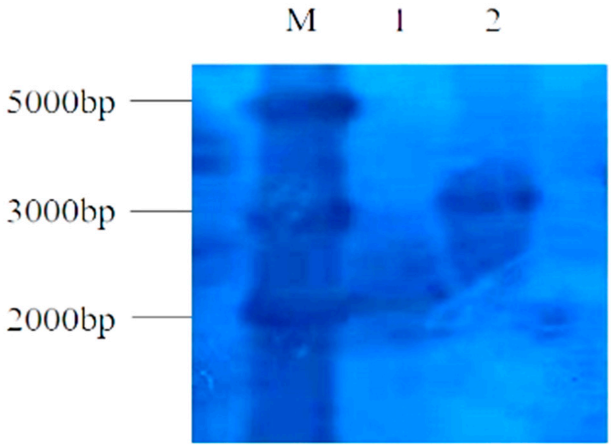

Figure S3. Results of Southern Blot X-ray. 1：HH103（*Xho* I），2：HH103Ω*NopT*＆*NopC*＆*NopL* (*Xho* I); M: DL 15000 DNA Ladder.

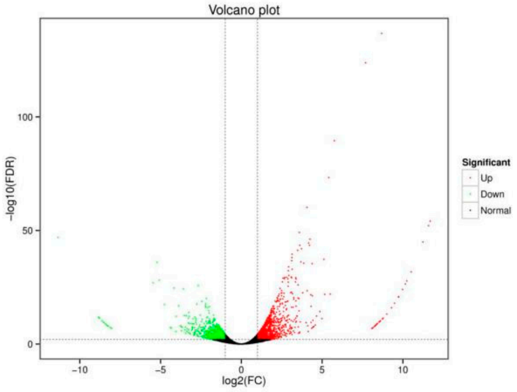

0.5 hpi $MgSO_4$ vs. HH103

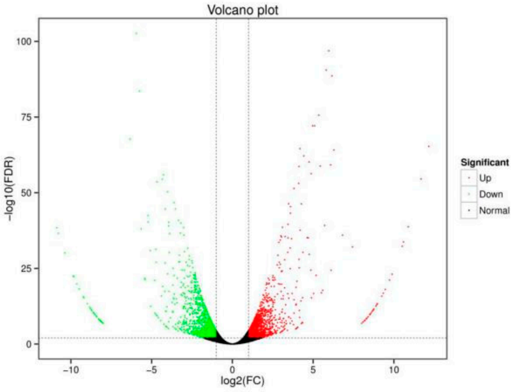

6 hpi $MgSO_4$ vs. HH103

Figure S4. The 0.5 hpi and 6 hpi volcano maps of the simulated treatment group ($MgSO_4$) and the inoculated rhizobium HH103 group.

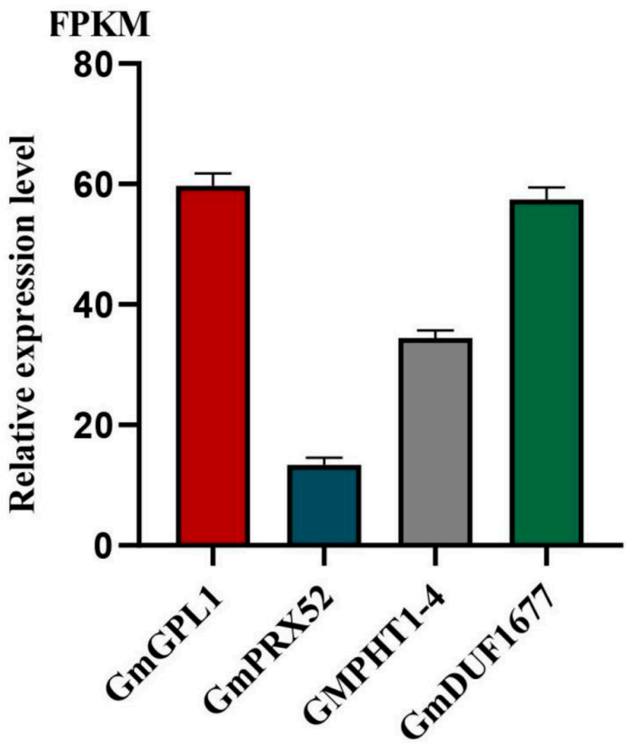

Figure S5. Expression level of candidate genes in soybean root.

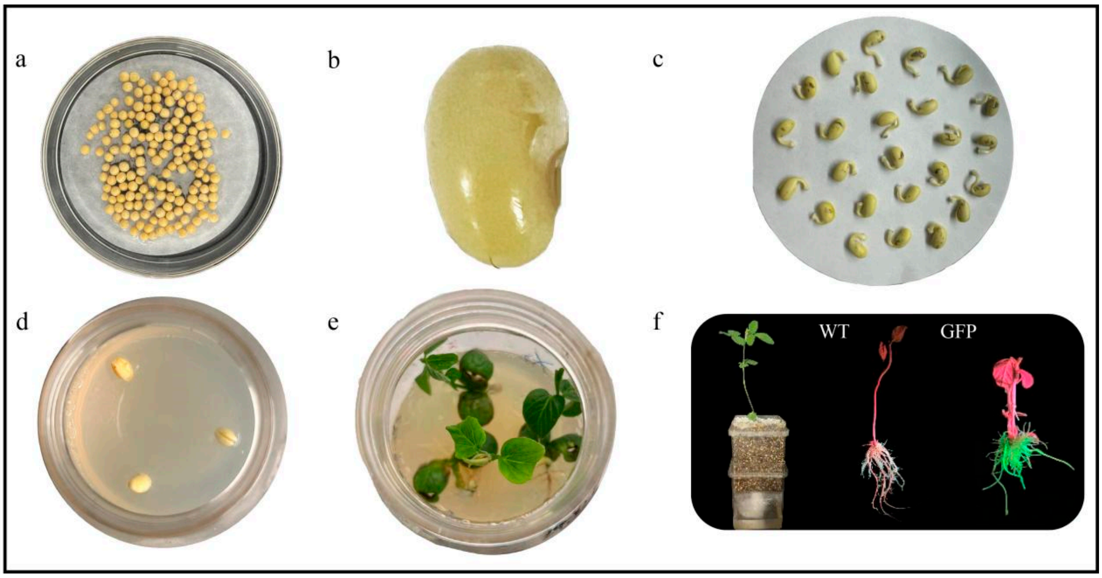

Figure S6. Hairy root transformation flow chart. (a) Seed germination by imbibition. （b） Remove the seed hypocotyl.（c）Swelling of growth points infected by bacterial solution. （d） Soybean inoculant takes root. （e）Recovery culture for 14 days. （f）Transfer to a double bowl and test positive.

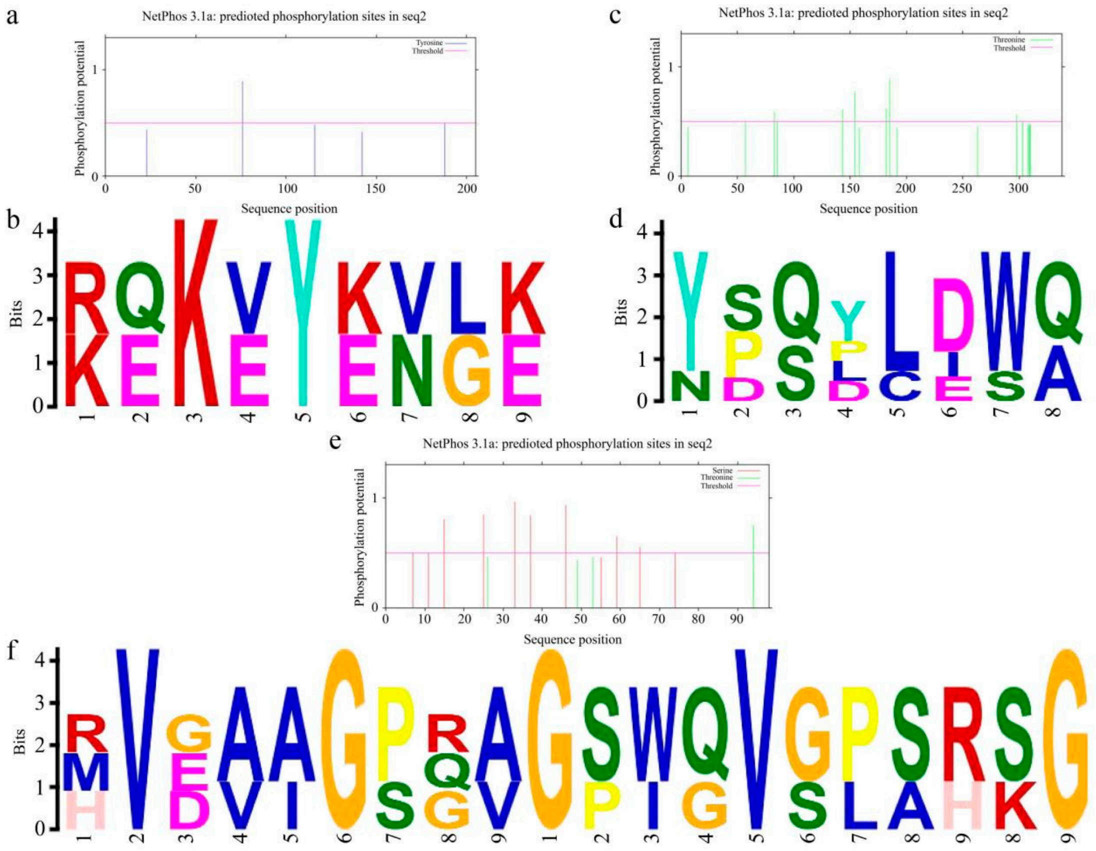

Figure S7. Prediction of NopT, NopL and NopC phosphorylation modification sites. (a-b) Prediction of NopT phosphorylation modification sites and corresponding modified amino acids. The larger the amino acid display, the higher the possibility of phosphorylation; (c-d) Prediction of NopL phosphorylation modification sites and corresponding modified amino acids; Prediction of (e-f) NopC phosphorylated modification sites and corresponding modified amino acids.

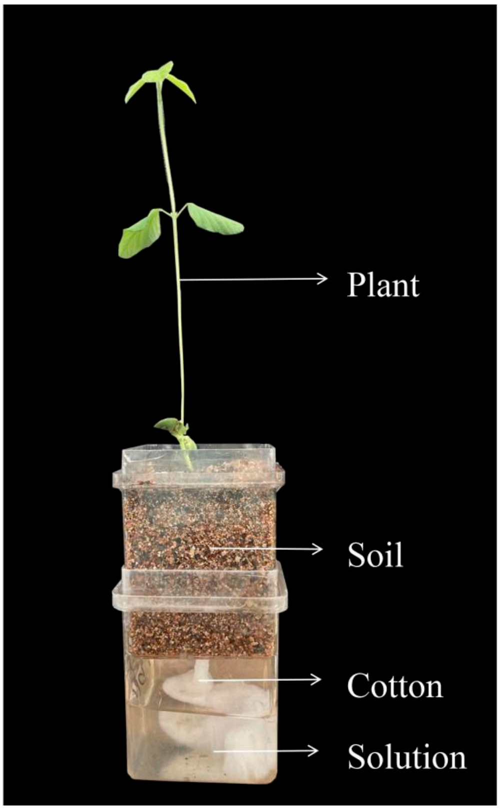

Figure S8. The double-layer pot apparatus,showing the solution, cotton rope, soil, and soybean plant.
