# Supplementary figures and images for: NopC/T/L Signal Crosstalk Gene GmPHT1-4
Source: Int J Mol Sci. 2023 Nov 20;24(22):16521. doi: 10.3390/ijms242216521 (PMC10671193; doi:10.3390/ijms242216521)

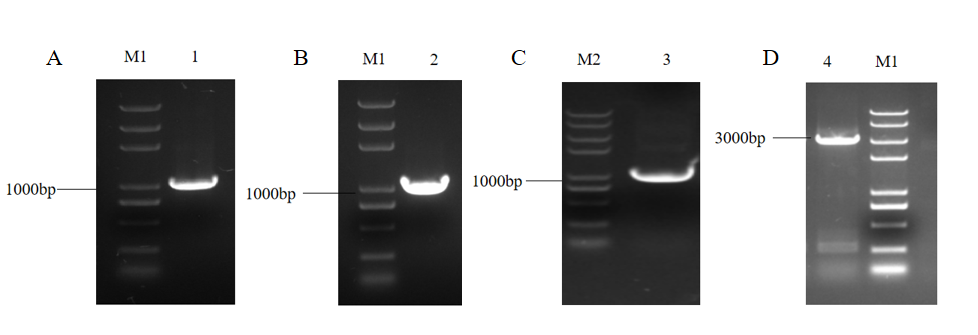

Supplement: Supplementary file 1 [file ijms-24-16521-s001.zip › Figure S1.jpg]

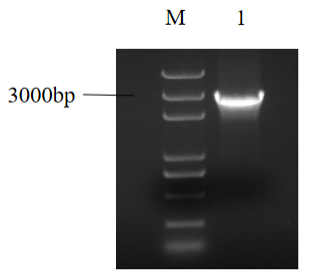

Supplement: Supplementary file 1 [file ijms-24-16521-s001.zip › Figure S2.jpg]

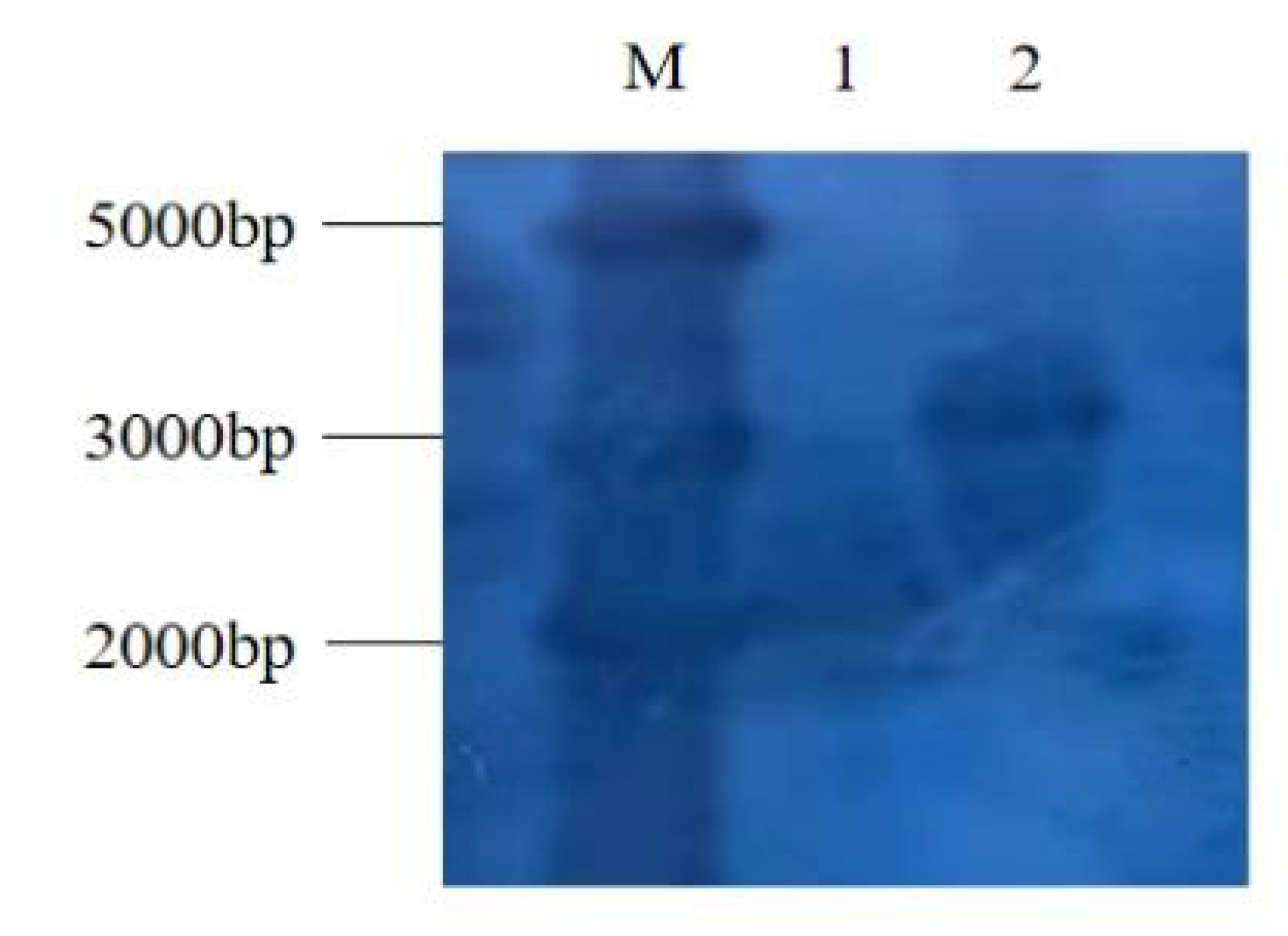

Supplement: Supplementary file 1 [file ijms-24-16521-s001.zip › Figure S3.jpg]

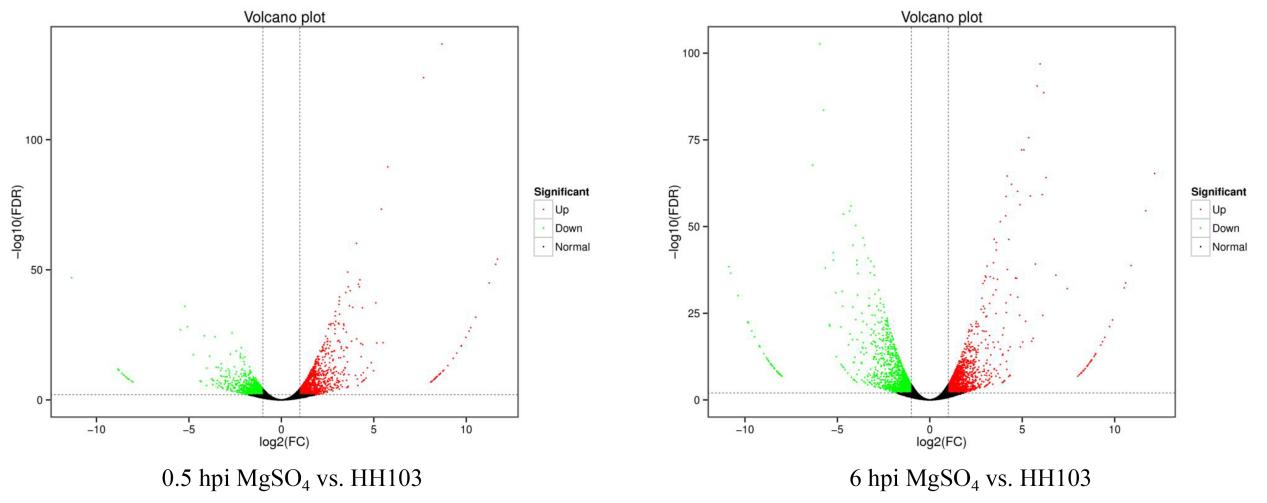

Supplement: Supplementary file 1 [file ijms-24-16521-s001.zip › Figure S4.jpg]

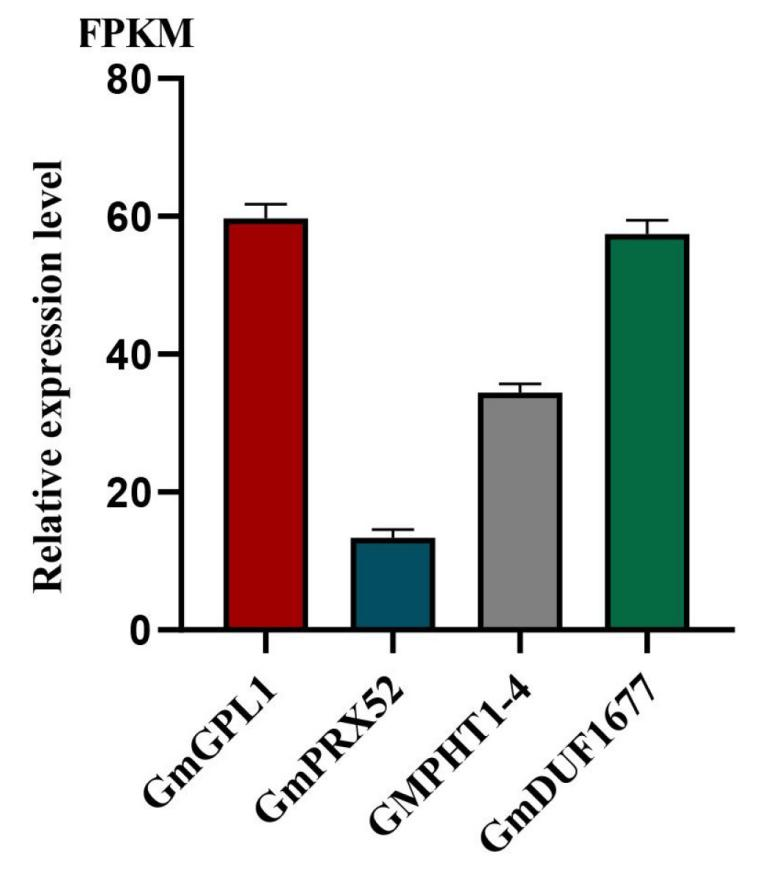

Supplement: Supplementary file 1 [file ijms-24-16521-s001.zip › Figure S5.jpg]

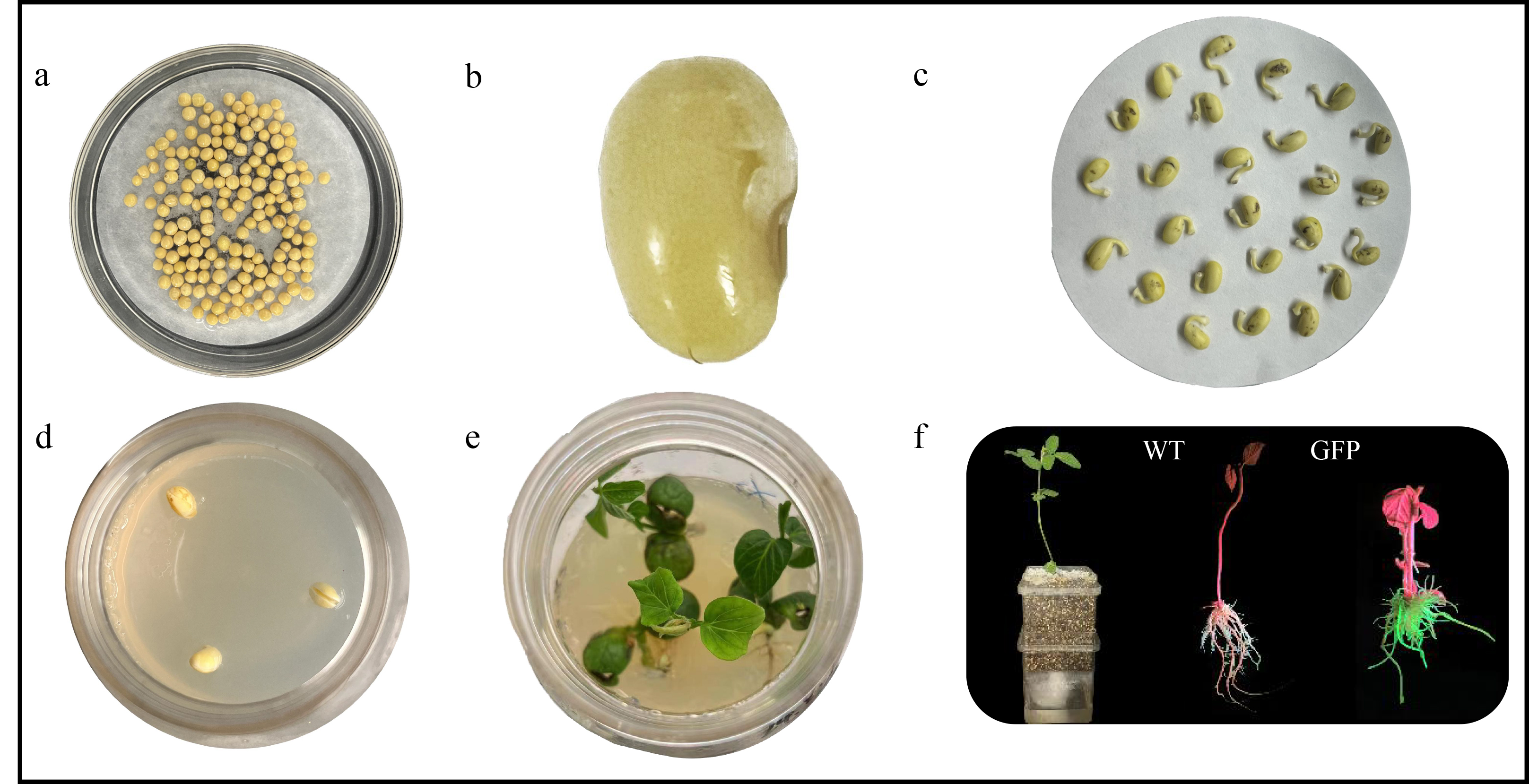

Supplement: Supplementary file 1 [file ijms-24-16521-s001.zip › Figure S6.jpg]

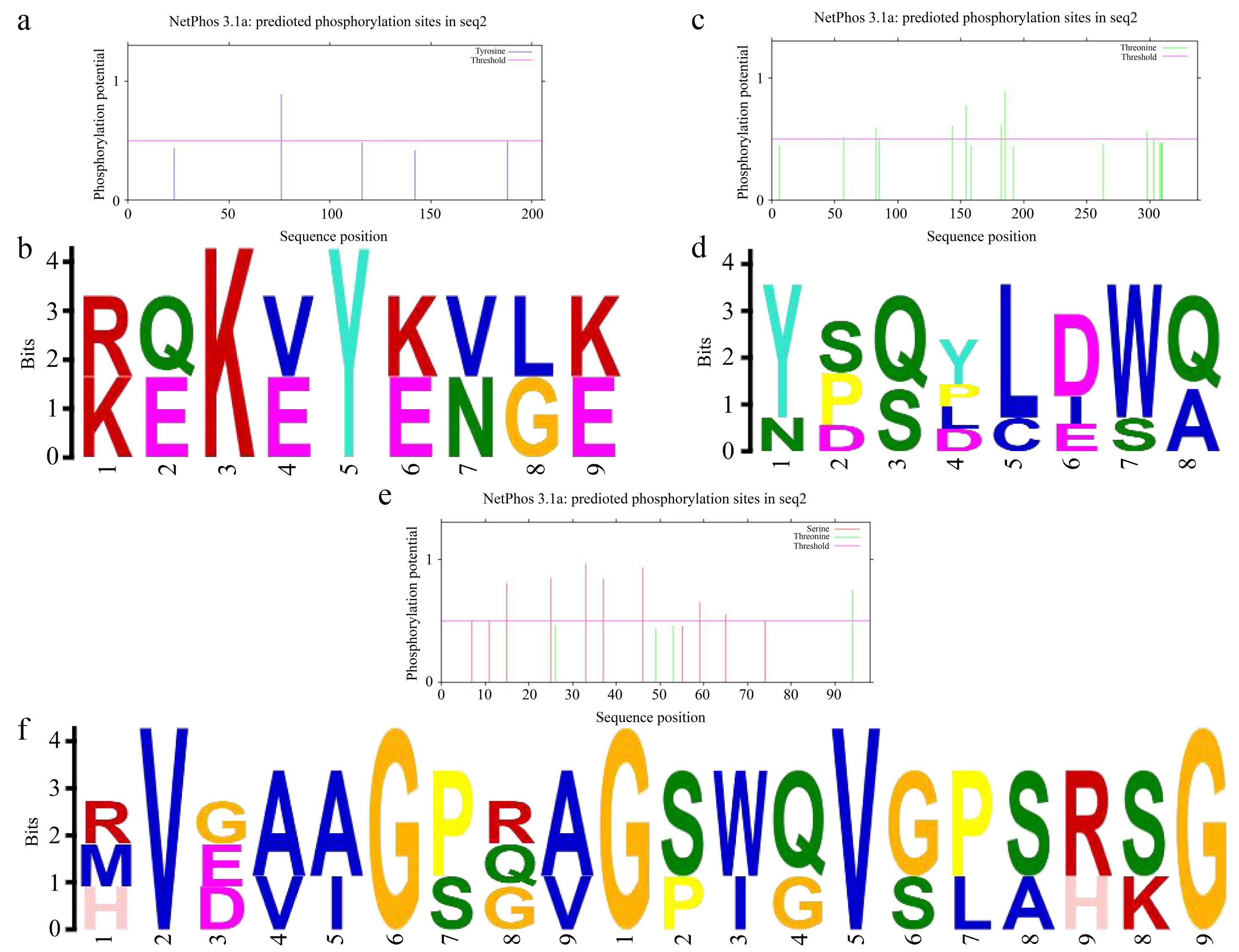

Supplement: Supplementary file 1 [file ijms-24-16521-s001.zip › Figure S7.jpg]

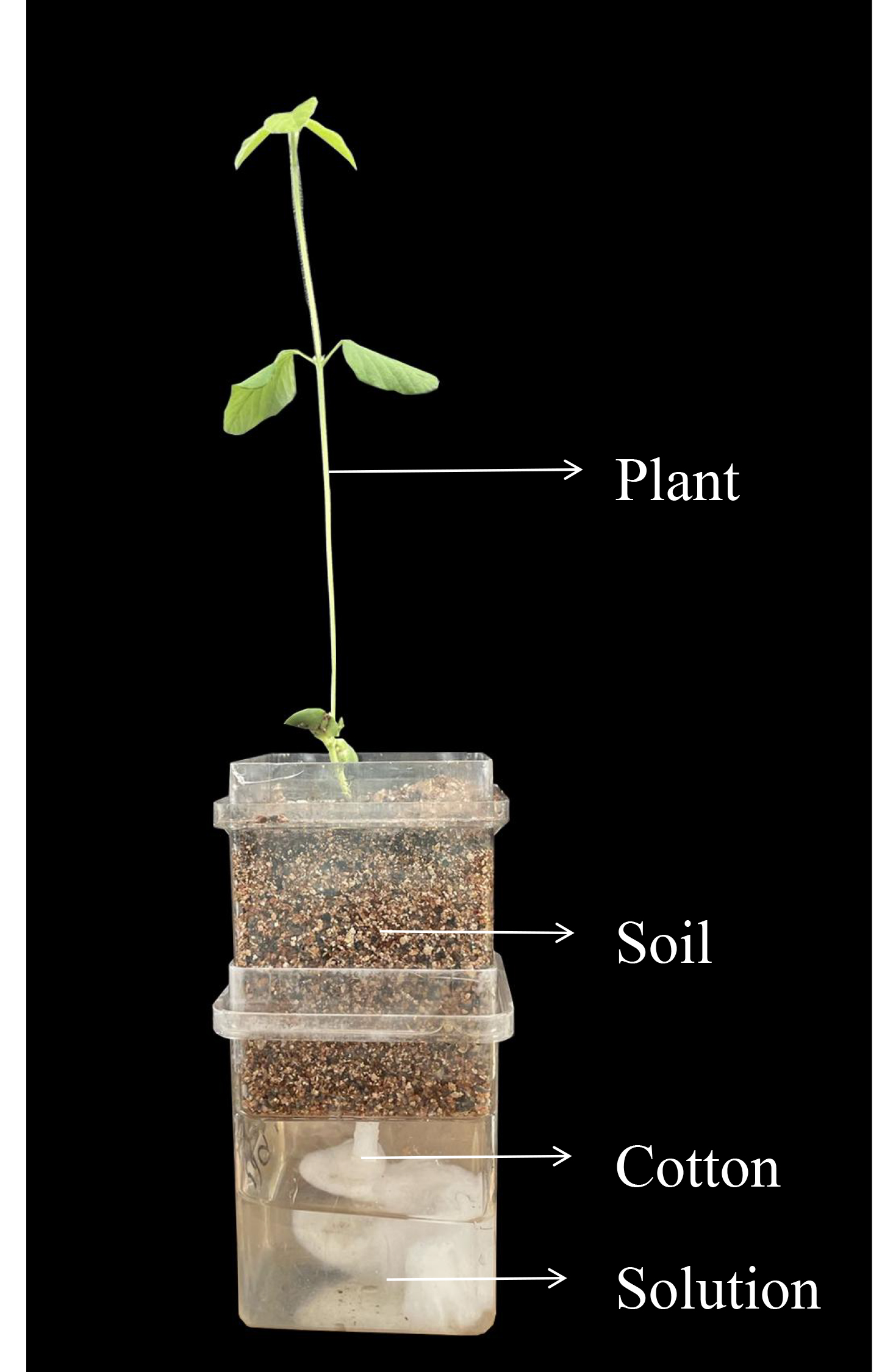

Supplement: Supplementary file 1 [file ijms-24-16521-s001.zip › Figure S8.jpg]
